# o-Vanillin Modulates Cell Phenotype and Extracellular Vesicles of Human Mesenchymal Stem Cells and Intervertebral Disc Cells

**DOI:** 10.3390/cells11223589

**Published:** 2022-11-13

**Authors:** Li Li, Kai Sheng, Matthew Mannarino, Peter Jarzem, Hosni Cherif, Lisbet Haglund

**Affiliations:** 1 Department of Surgery, McGill University, Montreal, QC H3G 1A4, Canada; 2 Shriners Hospital for Children, Montreal, QC H4A 0A9, Canada

**Keywords:** disc degeneration, cell therapy, trophic effect, hMSC differentiation, disc cell phenotype, extracellular vesicles, o-Vanillin, senolytic

## Abstract

Human mesenchymal stem cell (hMSC) and extracellular vesicle (EV) therapy is a promising treatment for discogenic low back pain (LBP). Although promising, major obstacles remain to be overcome. Cellular senescence reduces self-renewal and multipotent potentials, and the senescence-associated secretory phenotype creates an inflammatory environment negatively affecting tissue homeostasis. Reducing senescence could therefore improve regenerative approaches. Ortho-Vanillin (o-Vanillin) has senolytic activity and anti-inflammatory properties and could be a valuable supplement to MSC and EV therapy. Here, we used direct co-culture experiments to evaluate proteoglycan synthesis, inflammatory mediators, and senescent cells in the presence or absence of o-Vanillin. EV release and transfer between hMSCs and intervertebral disc cells (DCs) was examined, and the effect on hMSC differentiation and DC phenotype was evaluated in the presence and absence of o-Vanillin. This study demonstrates that o-Vanillin affects cell communication, enhances hMSC differentiation and improves DC phenotype. Co-cultures of DCs and hMSCs resulted in increased proteoglycan synthesis, a decreased number of senescent cells and decreased release of the cytokines IL6 and 8. Effects that were further enhanced by o-Vanillin. o-Vanillin profoundly increased EV release and/or uptake by hMSCs and DCs. DC markers were significantly upregulated in both cell types in response to conditioned media of o-Vanillin treated donor cells. Collectively, this study demonstrates that o-Vanillin affects hMSC and DC crosstalk and suggests that combining hMSCs and senolytic compounds may improve the outcome of cell supplementation and EV therapy for LBP.

## 1. Introduction

Low back pain (LBP) is the leading cause of disability worldwide with a lifetime prevalence exceeding 84% [1,2]. LBP-related medical costs are estimated between CAD 6–12 billion annually in Canada [3] and approximately USD 90 million in the United States in 2018 [4].

Intervertebral disc (IVD) degeneration is a major etiology of LBP [5]. Degenerating IVDs lose proteoglycan content, and healthy resident disc cells (DCs) [6]. They gradually lose disc height and mechanical properties and may herniate, which all result in pain. Human mesenchymal stem cell (hMSC) therapy is a promising therapeutic option to restore impaired IVDs [7]. Mesenchymal stem cells (MSCs) derived from human bone marrow are one of the most intensively studied cells for IVD regeneration [8,9]. They secrete trophic factors, such as anti-inflammatory cytokines and growth factors, that support resident DCs and have the potential to improve their phenotype. They also promote extracellular matrix (ECM) production [5]. A previous study showed that conditioned media (CM) of hMSCs enhanced viability and ECM production in DC pellet cultures [10]. Another study also illustrated that co-culture with IVD cells promotes monolayer hMSC differentiation toward a nucleus pulposus (NP)-like cell phenotype [11].

Although current hMSC therapy shows promising results in some preclinical and clinical studies [6], several obstacles such as stem cell exhaustion remain to be overcome [12]. Stem cell exhaustion can be induced by cellular senescence, which alters their self-renewal (proliferation) and multipotent (differentiation) potentials [12,13]. Senescent cells are protected from apoptosis and adopt a senescence-associated secretory phenotype [12,14]. Ortho-Vanillin (o-Vanillin) is a natural compound known for its anti-inflammatory properties in acute kidney injury and arthritis in rodents [15,16]. Our group previously reported that o-Vanillin in addition to its anti-inflammatory properties, has senolytic effects, sending senescent human DCs to apoptosis. It also increases cell proliferation of non-senescent cells from degenerate IVDs [17]. Studies evaluating gene networks affected by o-Vanillin showed that it affects networks involved in cell death and survival, cell cycle progression and connective tissue development and function [15,18,19,20,21]. o-Vanillin has also been studied in different types of cancers. It exhibited cytotoxicity against human melanoma cells in vitro and suppressed melanoma growth in mice [22]. In addition, o-Vanillin reduced glioma growth in murine brain slice cultures and inhibited glioma-induced proliferation of murine primary microglia [20]. In this study, we investigated o-Vanillin’s effects on hMSCs in direct cell–cell contact cultures of hMSCs and DCs from degenerate IVDs in three-dimensional (3D) pellet cultures and in indirect pellet culture where CM was transferred between the cell types. We hypothesized that o-Vanillin would remove senescent hMSCs and DCs thereby improving the microenvironment and trophic effects. The cumulative effects of o-Vanillin on IVD cells and hMSCs could significantly promote stem cell therapy in IVD repair.

## 2. Materials and Methods

### 2.1. The Tissue Collection

Degenerate disc tissue from consenting patients undergoing spinal surgery for LBP was collected according to the procedures approved by McGill University ethical review board (IRB#s A04-M53-08B and Tissue Biobank 2019–4896). Patient demographics are presented in Table 1. Disc tissue was processed by removing the outer annulus fibrosis (oAF) tissues from the NP and inner AF (iAF) tissues and disc cells were isolated as previously described [17,23]. Briefly, the combined NP and iAF tissues were washed, minced, and digested with type II collagenase (ThermoFisher Scientific, Toronto, ON, Canada) for 16 h at 37 °C. Isolated cells were re-suspended in Dulbecco’s Modified Eagle Medium (DMEM) (Sigma-Aldrich, Oakville, ON, Canada) with 2.25 g/L glucose supplemented with 10% (*v*/*v*) fetal bovine serum (FBS) (ThermoFisher Scientific, Toronto, ON, Canada), 100 µg/mL Primocin^TM^, 2 mM GlutaMAX^TM^ (ThermoFisher Scientific, Toronto, ON, Canada), and incubated at 37 °C with 5% CO_2_.

### 2.2. Cell Culture

Monolayer culture: Human-bone-marrow-derived MSCs were purchased from RoosterBio, Inc., (Frederick, MD, USA) (Cat. #MSC-001). One million cells (hMSCs or DCs) were seeded in T75 cell culture flasks (Sarstedt, TC Flask T75, Stand, Vent. Cap, Germany) and cultured in DMEM media (2.25 g/L glucose DMEM (ThermoFisher Scientific, Toronto, ON, Canada) supplemented with 10% (*v*/*v*) FBS, 2 mM GlutaMAX^TM^, and 0.25 mg/mL Gentamicin (ThermoFisher Scientific, Toronto, ON, Canada)) at 37 °C and 5% CO_2_. hMSCs were cultured in the same DMEM supplemented with recombinant human basic fibroblast growth factor 2 (Applied Biological Materials Inc., Richmond, BC, Canada) at 5 ng/mL during the expansion phase [24,25]. Culture media was changed twice a week and cells were used for pellet cultures within 1–2 passages.

Pellet culture: Three hundred thousand cells were pelleted with different cell proportions at the following combination ratios: 100% hMSCs, 100% DCs and DCs:hMSCs 1:1, 2:1, and 3:1. Pellets were cultured in 1 mL DMEM media (2.25 g/L glucose supplemented with 5% FBS, 50 μg/mL ascorbic acid, 2 mM GlutaMAX^TM^, and 0.25 mg/mL Gentamicin) at 37 °C and 5% CO_2_. Pellets were generated and left to mature for 2 days, following which they were treated for 4 days with 100 μM o-Vanillin (Sigma-Aldrich, Oakville, ON, Canada) or vehicle 0.01% (*v*/*v*) DMSO (Sigma-Aldrich, Oakville, ON, Canada) and then cultured for an additional 21 days with a media change in every 4 days. Media from the 21-day period were collected and stored at −20 °C until analyzed. The o-Vanillin concentration resulting in the highest senolytic effect has been determined in our previous work [17].

### 2.3. Histology Assessments

Cell pellets were washed with phosphate-buffered saline (PBS) (Sigma-Aldrich, Oakville, ON, Canada), fixed with 4% paraformaldehyde (Sigma-Aldrich, Oakville, ON, Canada) and prepared for cryopreservation according to our previous work [17]. Pellets were then embedded in Tissue-Plus™ Optimal Cutting Temperature Compound (Fisher Scientific, Canada), flash-frozen stored at −80 °C. Five-micrometer sections were prepared using a CryoStar NX70 cryostat (ThermoFisher Scientific, Toronto, ON, Canada) and thaw-mounted on Fisherbrand™ Superfrost™ Plus microscope slides (Fisher Scientific, Canada), and then stored at −20 °C for staining.

Safranin-O staining: Pellet sections were exposed to 50 °C for 30 min followed by a PBS rehydration at room temperature. Samples were then immersed in 0.1% (*w*/*v*) Safranin-O (Sigma-Aldrich, Oakville, ON, Canada) for 5 min at room temperature and rinsed with running tap water for 10 min. Dehydration was performed with 75% and 95% ethanol and samples were mounted with Permount^TM^ Mounting Medium (Fisher Scientific, Canada) and imaged using a Leica DMRB microscope (Leica Microsystems, Wetzlar, Germany) with a pre-fixed Olympus DP70 digital camera (Olympus Scientific Solutions, Tokyo, Japan) under visible light.

p16*^INK4a^* staining: Following incubation at 50 °C for 30 min, pellet sections were rehydrated in PBS-T (0.1% (*v*/*v*) Triton X-100, Sigma-Aldrich, Oakville, ON, Canada). Pellet sections were blocked with hydrogen peroxide, rinsed with 1X CINTec Wash Buffer (CINTec Kit, Roche), then blocked with PBS-T-Tween 20 (1% (*v*/*v*)) with 1% (*w*/*v*) bovine serum albumin (Sigma-Aldrich, Oakville, ON, Canada) and 1% (*v*/*v*) goat serum. Samples were exposed to p16*^INK4a^* antibody solution (50 mmol/L) (CINTec Kit, Roche) at 4 °C overnight. Detection of p16*^INK4a^* immunopositivity was performed using HRP/DAB Detection IHC Kit (ab64264, Abcam). Meyer’s hematoxylin (Sigma-Aldrich, Oakville, ON, Canada) was used as a nuclear counterstain. Samples were dehydrated with 75% and 95% ethanol and then mounted, imaged, and analyzed as described for Safranin-O staining.

### 2.4. Dimethylmethylene Blue (DMMB) Assay

Sulfated glycosaminoglycans (GAGs) released in the CM of hMSC and DC pellets was quantified using DMMB assays as previously described [17]. CM of hMSC or DC pellets was pooled from the respective cell type and treatment. A serial dilution of chondroitin sulfate (C9819, Sigma-Aldrich, Oakville, ON, Canada) was used to generate the standard curve. DMMB dye was added to samples followed by an immediate measurement of the absorbance at 405 nm using a TECAN Infinite M200 PRO plate reader with i-control 1.9 Magellan software (TECAN, Männedorf, Switzerland). Data of co-culture groups were normalized with the formular (Normalized GAG concentration = GAG concentration/DC percentage).

### 2.5. Enzyme-Linked Immunosorbent Assay (ELISA)

Protein concentrations of inflammatory cytokines IL-6 and IL-8 present in the collected culture media were measured using ELISA kits (RayBiotech, Peachtree Corners, GA, USA) according to the manufacturer’s instructions. Post-treatment CM of hMSC or DC pellets were pooled. Absorbance was measured using a TECAN Infinite M200 PRO plate reader with i-control 1.9 Magellan software (TECAN, Männedorf, Switzerland). Protein levels of untreated and o-Vanillin-treated samples were compared.

### 2.6. Plasma Membrane Labeling and Confocal Microscope

hMSCs and DCs were released with trypsin, collected, resuspended and the plasma membrane was labeled with a Vybrant™ DiI cell-labeling solution (V22885, ThermoFisher Scientific, Toronto, ON, Canada) according to the manufacturer’s instructions. One million labeled or unlabeled cells were seeded on 24 mm × 50 mm sterile cover glass (Fisher Scientific, Canada) which were placed in a 10 cm-cell culture dish (Sarstedt, Germany). Cells were cultured in DMEM media with or without o-Vanillin and successful labeling was confirmed (Figure S1A). Unlabeled cells were also cultured in the same conditions as recipient and control samples. CM was centrifuged at 1500 rpm for 5 min to remove debris and hMSC CM was applied to unlabeled DCs and vice versa (Figure S1A). After a 7-day incubation, cell nuclear DNA was stained with NucBlue^TM^ reagent (ThermoFisher Scientific, Toronto, ON, Canada) and extracellular vesicle (EV) uptake was examined under a LSM780 confocal microscope and ZEN software (ZEISS, Jena, Germany). The florescent intensity was measured using Fiji Image J software [26]. One million hMSCs and DCs (with or without (Figure S4) DiI labeling) were used to generate EVs from untreated (vehicle) or treated (o-Vanillin) cells (Figure S1B). Following the treatment of 4 days, pellets were incubated for another 4 days in DMEM media and CM were collected and cleaned by centrifugation as described above. The structure and size distribution (manually counting) of the EVs in CM was examined using an LSM780 confocal microscope and ZEN software (ZEISS, Jena, Germany) (Figure S1B).

### 2.7. Nano-Flow Cytometry

One million of DiI-labeled hMSCs and DCs were exposed to vehicle (0.01% (*v*/*v*) DMSO) or 100 µM o-Vanillin for 4 days. The cell culture media were changed to fresh DMEM without o-Vanillin on days 5 and 8. CM of days 5 and 8 were collected, and debris was removed by centrifugation. CM samples and standard cell culture media (set as control) were diluted with filtered PBS at 1:80 and prepared for nano-flow cytometry. Sample acquisition was performed using a CytoFLEX flow cytometer (Model No. A00-1-1102) and a CytExpert software (BECKMAN COULTER Life Science, USA) according to the manufacturer’s guidelines. Briefly, the acquisition was set to 2 min with a 10 µL/min flow rate, < 2% abort rate, and < 10,000 event/second speed to ensure a good resolution. ApogeeMix calibration beads (#1493, APOGEE flow systems, UK) were used as size references. The total event window was set to 110–1300 nm. The area of interest was gated using cell culture media that had not been in contact with cells. The data were analyzed using the CytExpert software and particles from the DMEM media were subtracted.

### 2.8. Real-Time Quantitative Polymerase Chain Reaction (qPCR)

Cell pellets were classified into CM provider and recipient groups as described in Figure S2. The CM providers were incubated with DMEM supplemented with vehicle 0.01% (*v*/*v*) DMSO or 100 µM o-Vanillin for 4 days, were then cultured in DMEM without o-Vanillin for 21 days, with media change every 4 days. Media from the provider group was transferred to the recipient group: from hMSC to DC pellets and from DC to hMSC pellets (Figure S2). CM was transferred to recipient pellets every 4 days (the 4-day treatment media were discarded). Pellets were paired from the first to the last transfer of the 21-day culture period (Figure S2).

Pellet samples were lysed in TRIzol^TM^ reagent (ThermoFisher Scientific, Toronto, ON, Canada) and RNA was extracted according to the manufacturer’s instructions as previously described [17,27]. Extracted RNA was converted to cDNA with a High-Capacity RNA-to-cDNA™ Kit (ThermoFisher Scientific, Toronto, ON, Canada) using Applied Biosystems^®^ Veriti^®^ 96-Well Fast Thermal Cycler (ThermoFisher Scientific, Toronto, ON, Canada). qPCR was performed in PROGENE^®^ 96-Well Half-Skirt ABI Fast PCR Plates (UltiDent Scientific, Canada) with PROGENE^®^ Adhesive Routine PCR Sealing Film (UltiDent Scientific, Canada) using an Applied Biosystems^®^ StepOnePlus™ Real-Time PCR System (ThermoFisher Scientific, Toronto, ON, Canada) with PowerUp™ SYBR™ Green Master Mix (ThermoFisher Scientific, Toronto, ON, Canada). Primers of hMSC and NP markers and a housekeeping gene are presented in Table 2. The melting curves were examined to exclude the risks of false amplifications. The data were normalized to the reference gene (*beta-ACTIN*) and presented in fold-changes calculated with the 2^−ΔΔCt^ method [28].

### 2.9. Statistical Analysis and Illustrations

Data were analyzed using GraphPad Prism version 9.3.1(350) (GraphPad, La Jolla, CA, USA). A D’Agostino-Pearson omnibus K2 normality test was conducted for data of all experiments. All the data are not inconsistent with a Gaussian distribution. Two-tailed Student’s *t*-test (paired or unpaired) and one-way ANOVA were used for data analysis. All assessments were conducted with three or more independent experiments, indicated by “*n*” in the figure legends. Each independent experiment was conducted with cells from one donor and three or more technical replicates from the same donor. The statistically significant difference was set at *p*-value < 0.05. Data are presented as mean ± SEM. The graphical abstract and schematic illustrations (Figure S1 and S2) were created with BioRender.com (accessed on 5 November 2022, publication license numbers: #NK24M0S0TM, #CK24M0R1BI, #SO24M0RVZ0, and #OG24M0RNS5).

## 3. Results

### 3.1. GAG Deposition and Release in DCs:hMSCs Co-Cultures

**Figure 1:**
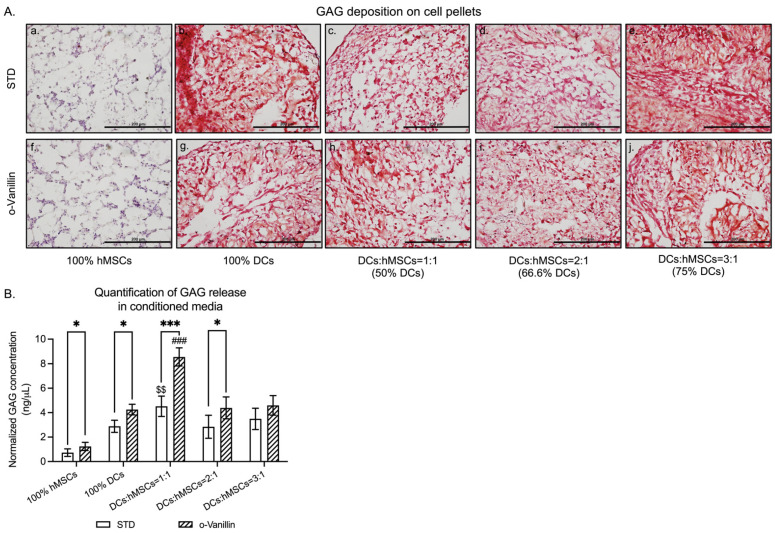
Assessments of sulfated glycosaminoglycan (GAG) synthesis in cell pellets of human mesenchymal stem cells (hMSCs), human disc cells (DCs), and co-cultures (1:1, 2:1, and 3:1 (DCs:hMSCs)) with and without ortho-Vanillin (o-Vanillin) treatment. (**A**) Representative images showing the GAG deposition in 100% hMSCs, 100% DCs, and co-cultures as assessed by Safranin-O staining. Scale bars: 200 μm. *n* = 4. (**B**) Evaluation of GAG release in the CM of 100% hMSC, 100% DC, and co-cultures as detected by DMMB assay. *n* = 5. Values are presented as mean ± SEM. * and *** indicate a statistically significant change assessed by a paired *t*-test: *p* < 0.05 and *p* < 0.001. $$ indicates a statistically significant assessment by a one-way ANOVA among untreated samples: *p* < 0.01. ### indicates a statistically significant assessment by a one-way ANOVA among o-Vanillin-treated samples: *p* < 0.001. Additionally, see Figure S3.

To evaluate the optimal ratio of DCs:hMSCs co-cultures, GAG production was measured in the presence or absence of o-Vanillin. We evaluated three ratios, 1:1, 2:1 and 3:1 of DCs:hMSCs. An optimal concentration of o-Vanillin for DCs has previously been determined; here we verified that this concentration has no cytotoxicity on hMSCs (Figure S3A). Safranin-O staining did not detect any GAG deposition in the untreated 100% hMSC pellets (Figure 1(A-a)). The untreated 100% DC pellets (Figure 1(A-b)) showed a similar amount of GAG compared to that of the untreated 3:1 (75% DCs) cultures (Figure 1(A-e)). With 50% DCs in the pellets, the untreated 1:1 group (Figure 1(A-c)) had a similar amount of GAG compared to that of the 2:1 (66.6% DCs) cultures (Figure 1(A-d)), but less than that of the 3:1 cultures (Figure 1(A-e)). Similarly, no GAG deposition was detected in the o-Vanillin-treated 100% hMSC pellets (Figure 1(A-f)). The o-Vanillin-treated 100% DC pellets (Figure 1(A-g)) had a similar amount of GAG compared to that of the o-Vanillin-treated 3:1 (75% DCs) cultures (Figure 1(A-j)). However, o-Vanillin-treated 1:1 (50% DCs) group (Figure 1(A-h)) showed more GAG than that of the o-Vanillin-treated 2:1 (66.6% DCs) cultures (Figure 1(A-i)) with a similar amount of GAG compared to that of the o-Vanillin-treated 3:1 cultures (Figure 1(A-j)).

The GAG concentration in the CM was measured and normalized to 3 × 10^5^ DCs. The GAG concentration of untreated 1:1 co-cultures (4.52 ± 0.83 ng/µL) was 55.17% higher than that of untreated 100% DCs (2.88 ± 0.50 ng/µL) and significantly higher than any other untreated groups (*p* = 0.0070) (100% hMSC cultures: 0.73 ± 0.31 ng/µL; 2:1 cultures: 2.84 ± 0.94 ng/µL; and 3:1 cultures: 3.49 ± 0.87 ng/µL) (Figure 1B). The accumulated GAG concentration in o-Vanillin-treated 1:1 co-cultures (8.56 ± 0.73 ng/µL) was 47.67% higher (*p* = 0.0006) than that of untreated 1:1 co-cultures and significantly greater than any other o-Vanillin-treated groups (*p* = 0.0001) (100% hMSC cultures: 1.24 ± 0.33 ng/µL; 100% DC cultures: 4.25 ± 0.44 ng/µL; 2:1 cultures: 4.39 ± 0.89 ng/µL; and 3:1 cultures: 4.59 ± 0.80 ng/µL) (Figure 1B). In addition, o-Vanillin significantly increased GAG release in 100% hMSC (*p* = 0.0386), 100% DC (*p* = 0.0202), and 2:1 (*p* = 0.0426) cultures. There was no significant difference in total DNA between the groups following 21 days of pellet culture (Figure S3B,C).

Taken together, these data indicate that the co-culture combination of 1:1 (DCs:hMSCs) resulted in the most robust GAG production, which was significantly improved by o-Vanillin treatment. Therefore, the subsequent experiments were performed with the co-culture ratio 1:1.

### 3.2. Presence of Senescent Cells and Inflammatory Cytokine Release in DCs:hMSCs Co-Cultures

**Figure 2:**
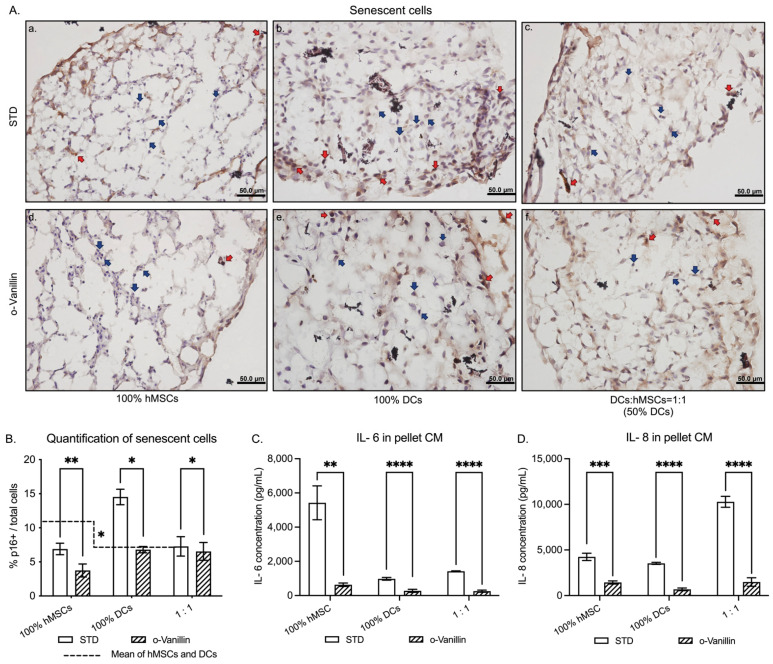
Assessments of senescent cells and inflammatory cytokine release in CM of hMSCs, DCs, and co-cultures (1:1 (DCs:hMSCs)) with and without o-Vanillin treatment. (**A**) Representative images showing immunostaining of p16*^INK4a^* positive cells (red arrows) and negative cells (blue arrows). Scale bars: 50 μm. *n* = 4. (**B**) Quantification of p16*^INK4a^* positive cells on hMSCs, DCs, and 1:1 co-culture pellets with or without o-Vanillin treatment. *n* = 4. (**C**) Effect of o-Vanillin on IL-6 production in the CM of 100% hMSC, 100% DC, and 1:1 co-culture pellets. *n* = 6. (**D**) Effect of o-Vanillin on IL-8 production in the CM of 100% hMSC, 100% DC, and 1:1 co-culture pellets. *n* = 6. Values are presented as mean ± SEM. *, **, ***, and **** indicate a statistically significant change assessed by a paired *t*-test: *p* < 0.05, *p* < 0.01, *p* < 0.001, and *p* < 0.0001, respectively.

Senescent cells accumulate in degenerating disc tissues and aging stem cells. The number of senescent cells in pellet cultures was evaluated using p16*^INK4a^* staining (Figure 2A). The untreated 100% hMSC and 100% DC pellets presented 6.90% ± 0.84 and 14.53% ± 1.13 p16*^INK4a^* positive senescent cells (Figure 2B). There was a 32.03% (*p* = 0.0121) reduction in senescent cells in untreated co-cultures (7.28% ± 1.43) compared to the mean of each cell type alone. When treated with o-Vanillin, the number of senescent cells further decreased to 3.74% ± 0.96 (*p* = 0.0064) in 100% hMSCs and to 6.79% ± 0.46 (*p* = 0.0121) in 100% DCs and to 6.54% ± 1.30 (*p* = 0.0126) in co-cultures. Together the results demonstrate that co-culture of hMSCs and DCs (1:1) result in a lower total number of senescent cells and that o-Vanillin treatment reduced both senescent hMSCs and DCs.

The release of inflammatory cytokines IL-6 and IL-8 was measured in the CM by ELISA. IL-6 concentrations decreased by 88.36% (*p* = 0.0060), 71.78% (*p* < 0.0001), and 81.91% (*p* < 0.0001), respectively, in o-Vanillin-treated 100% hMSCs (from 5422 ± 99 to 631 ± 101 pg/mL), 100% DCs (from 978 ± 78 to 276 ± 88 pg/mL), and 1:1 co-cultures (from 1421 ± 22 to 257 ± 61 pg/mL) (Figure 2C). While IL-8 concentrations decreased by 66.19% (*p* = 0.0001), 80.54% (*p* < 0.0001), and 85.55% (*p* < 0.0001), respectively, in o-Vanillin-treated 100% hMSC (from 4247 ± 401 to 1436 ± 170 pg/mL), 100% DC (from 3526 ± 112 to 686 ± 145 pg/mL), and 1:1 co-cultures (from 10,278 ± 601 to 1485 ± 470 pg/mL) (Figure 2D).

### 3.3. Extracellular Vesicle Generation and Release

**Figure 3:**
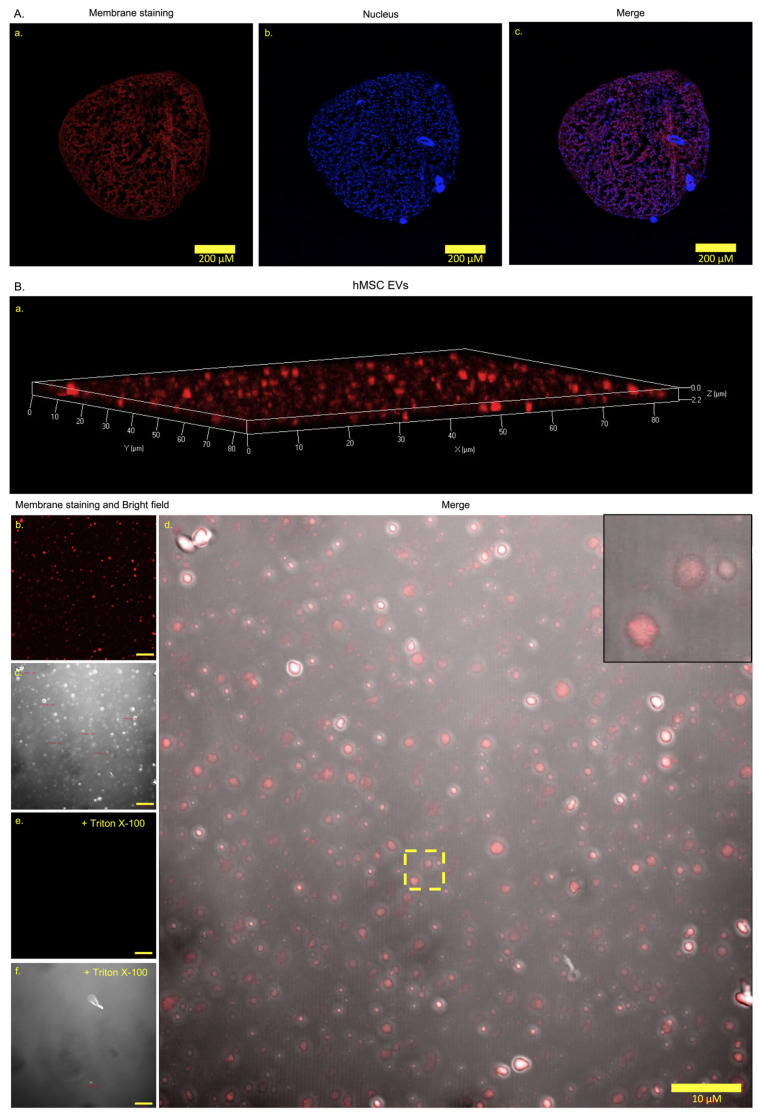

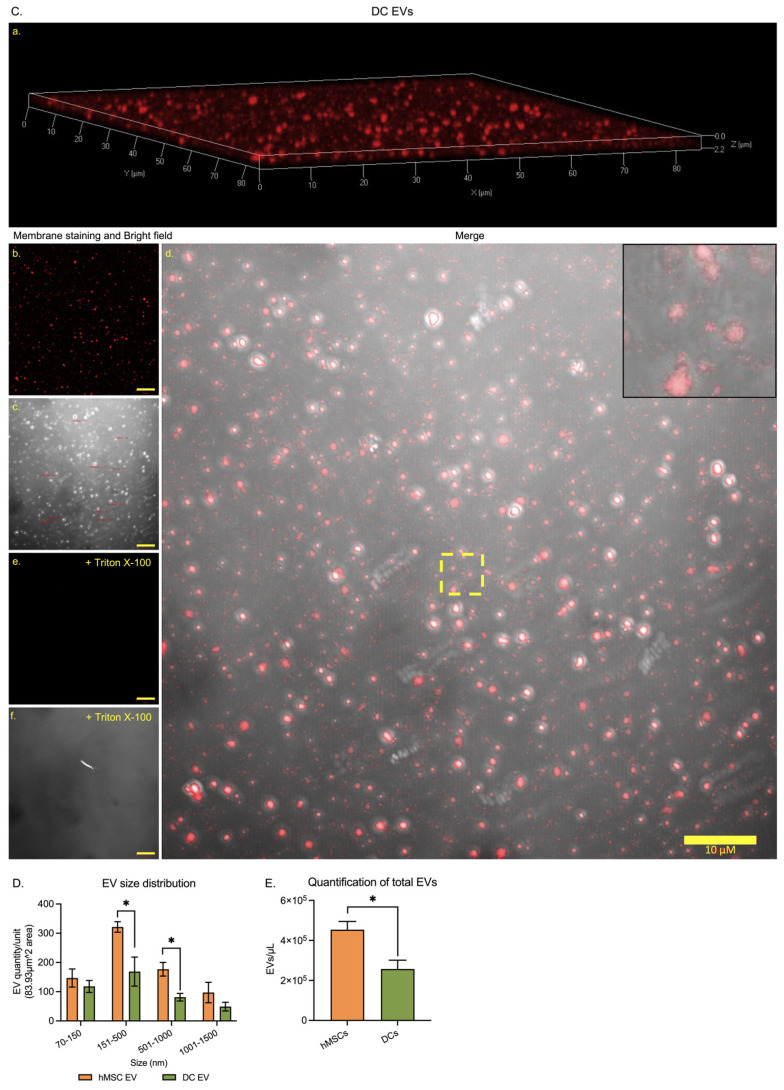
EV production by DiI-labeled hMSCs and DCs. (**A**) Representative images showing plasma membrane (red) (**A-a**), nuclear (blue) (**A-b**), and merge staining (**A-c**) of co-cultured unlabeled DCs and DiI(red)-labeled hMSCs. Scale bars: 200 μm. *n* = 4. (**B**,**C**) Representative images show the 3D EV structures (**B-a**,**C-a**), membrane signal (**B-b**,**C-b**), the size variation of EVs in bright field (**B-c**,**C-c**), the merge of the membrane signal and the EV structures (**B-d**,**C-d**), the loss of signal after exposure to detergent (**B-e**,**C-e**), and the loss of EV structures (**B-f**,**C-f**) when exposed to detergent of EVs generated by labeled hMSCs (**B**) and DCs (**C**). Scale bars: 10 µm. *n* = 3. (**D**). Manually counting under a confocal microscope illustrates the size distribution of EVs derived from hMSCs and DCs demonstrated in (**B**) and (**C**). *n* = 3. (**E**). Quantification of the total numbers of EVs generated by labeled hMSCs and DCs using nano-flow cytometry. The total event window is set to 110–1300 nm using ApogeeMix calibration beads. *n* = 4. Values are presented as mean ± SEM. * indicates a statistically significant change assessed by an unpaired Welch’s *t-*test in (**D**,**E**): *p* < 0.05. Additionally, see Figure S4.

To evaluate how cell phenotype is affected by co-culture, hMSCs were labeled with a red-lipophilic-fluorescent-membrane-dye DiI. Labeled hMSCs were cultured with unlabeled DCs (1:1 DCs:hMSCs). The goal was to separate red from unlabeled cells after co-culture. However, all cells were red after a 21-day culture period (Figure 3(A-a–c)). This revealed that separation of two cell types would not be possible.

EV transfer is a known cell communication process. If EVs are released and transferred, this could result in all cells appearing red after co-culture. To evaluate this, CM from labeled hMSCs and DCs were collected and analyzed. CM from unlabeled hMSCs and DCs were collected and analyzed as references (Figure S4). Vesicles were generated by both hMSCs Figure 3(B-a–d) and Figure S4) and DCs (Figure 3(C-a–d) and Figure S4). Vesicles generated by labeled cells appeared red (Figure 3(B-b) and (C-b)). In addition, exposure to detergent removed all vesicular structures and the red signal (Figure 3(B-e,f) and (C-e,f)). Confocal microscopy was then used to determine vesicle distributions in set size ranges. The size of exosomes is less than 150 nm [33] and the size of microvesicles is 150–1000 nm [34] in diameter. As the resolution of LSM780 confocal microscope is 70 nm, four size ranges were set: 70–150 nm, 151–500 nm, 501–1000 nm, and 1001–1500 nm. Higher number of EVs were observed per unit (83.93 µm x 83.93 µm area) in hMSCs compared to those in DCs in the 151–500 nm (321.5 ± 17.86/unit and 168.8 ± 49.76/unit, *p* = 0.0447) and the 501–1000 nm (176.8 ± 23.20/unit and 81.0 ± 13.00/unit, *p* = 0.0227) groups (Figure 3D). No significant quantitative difference was observed between EVs derived from hMSCs and DCs in the 70–150 nm (146.8 ± 30.91/unit and 118.0 ± 20.50/unit, *p* = 0.4803) and the 1001–1500 nm (97.17 ± 35.05/unit and 49.0 ± 14.74/unit, *p* = 0.2740) groups (Figure 3D). These indicate that hMSCs generated larger EVs compared to those of DCs in the range of 151–1000 nm (Figure 3D). The quantity of EVs generated by hMSCs and DCs was also evaluated using nano-flow cytometry. The observation window was set to 110–1300 nm to cover the range of 151–1000 nm, from which significantly different EV quantities of hMSCs and DCs were observed with manually counting. The total EVs generated by hMSCs (453,809 ± 41,183/µL) was significantly greater (*p* = 0.0170) than those generated by DCs (257,473 ± 43,609/µL) (Figure 3E).

### 3.4. Extracellular Vesicle Transfer and Uptake

**Figure 4:**
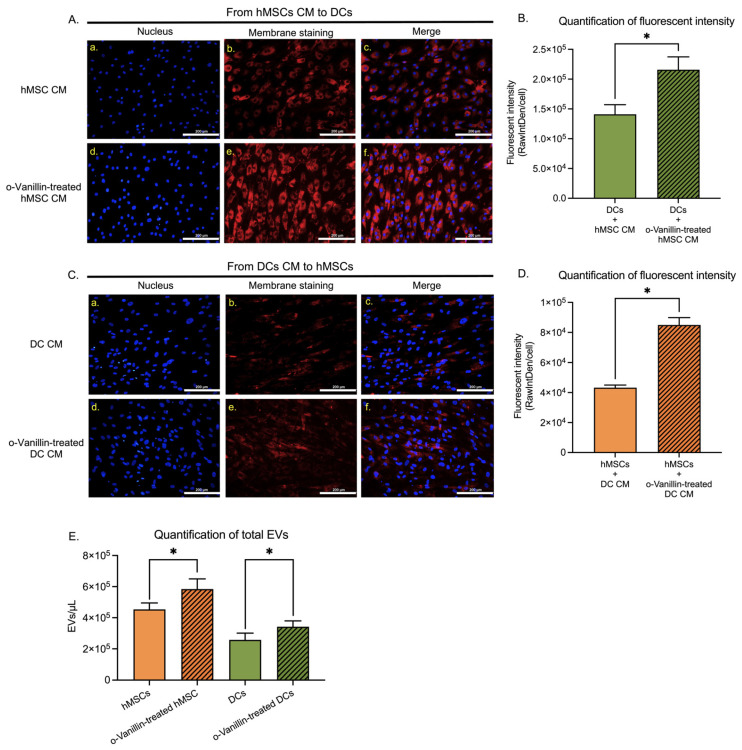
Transfer and uptake of EVs by hMSCs and DCs via CM. (**A**) Representative images showing nuclear (blue) (**A-a**), plasma membrane (red) (**A-b**), and merge staining (**A-c**) of DCs cultured in the CM of red-labeled untreated hMSCs (**A-a–c**) and o-Vanillin-treated hMSCs (**A-d–f**). Scale bars: 200 μm. *n* = 3. (**B**) Quantification of cellular fluorescence intensity in the two groups of DCs exposed to the CM of labeled untreated and o-Vanillin-treated hMSCs. *n* = 3. (**C**) Photomicrographs showing nuclear, plasma membrane, and merge staining of hMSCs cultured in the CM of labeled untreated (**C-a–c**) and o-Vanillin-treated (**C-d–f**) DCs. Scale bars: 200 μm. *n* = 3. (**D**) Quantification of cellular fluorescent intensity of hMSCs cultured in the CM of labeled untreated and o-Vanillin-treated DCs. *n* = 3. (**E**) Quantification of the total numbers of EVs generated by labeled o-Vanillin-treated hMSCs and DCs using nano-flow cytometry. The total event window is set to 110–1300 nm using ApogeeMix calibration beads. *n* = 4. Values are presented as mean ± SEM. * indicates a statistically significant change assessed by a paired *t-*test in (**B**,**D**,**E**): *p* < 0.05. Additionally, see Figure S5.

To examine EV communication between hMSCs and DCs in the presence or absence of o-Vanillin, CM from labeled host cells was collected and applied to the recipient hMSCs or DCs. After seven days of incubation with CM from the host cells, punctuated red fluorescence was observed in the recipient cells (Figure 4(A,C)). These results indicated that hMSC-derived EVs were taken up by DCs from the CM (Figure 4(A-a–c)). An increase in the red signal (Figure 4(A-d–f)) was observed when CM from o-Vanillin-treated hMSCs was applied to DCs. Cellular fluorescent intensity significantly increased from 0.1839 ± 0.0753 (Figure S5A) in cells cultured in standard media (Figure S5(B-a–c)) to 141,074 ± 15,984 (*p* = 0.0126) (Figure 4B) in DCs exposed to CM of labeled hMSCs, the signal was further significantly increased to 215,797 ± 21,509 (*p* = 0.0236) (Figure 4B) when exposed to CM from o-Vanillin-treated hMSCs. Similarly, DC-derived EVs were taken up by hMSCs, when hMSCs were exposed to DC CM (Figure 4(C-a–c)), confirming EV transfer from DCs to hMSCs. An increase in the red signal (Figure 4(C-d–f)) was observed when CM from o-Vanillin-treated DCs was applied to hMSCs, confirming o-Vanillin also promoted EV transfer from DCs to hMSCs. Cellular fluorescent intensity significantly increased from 0.1442 ± 0.0140 (Figure S5C) in cells cultured in standard media (Figure S5(D-a–c)) to 43,275 ± 1760 (*p* = 0.0016) (Figure 4D) in hMSCs exposed to CM of labeled DCs, the signal was further significantly increased to 84,993 ± 4849 (*p* = 0.0242) (Figure 4D) when exposed to CM from o-Vanillin-treated DCs. As host cells and EV providers, red hMSCs and DCs were labeled at a similar fluorescent intensity level with and without o-Vanillin treatment (Figure S5(E–H)). Of note, the EV transfer efficiency from hMSCs to DCs (Figure 4B) was higher than that from DCs to hMSCs (Figure 4D).

The quantity and size of EVs generated by o-Vanillin-treated hMSCs and DCs were also evaluated. The total amount of EVs was measured using nano-flow cytometry in the range of 110–1300 nm. o-Vanillin significantly increased the total amount of EVs from hMSCs by 28.81% (from 453,809 ± 41,183/µL to 584,557 ± 65,337/µL, *p* = 0.0250) and from DCs by 32.96% (from 257,473 ± 43,609/µL to 342,337 ± 37,346/µL, *p* = 0.0113) (Figure 4E).

### 3.5. hMSC Differentiation and DC Phenotype in Response to CM

**Figure 5:**
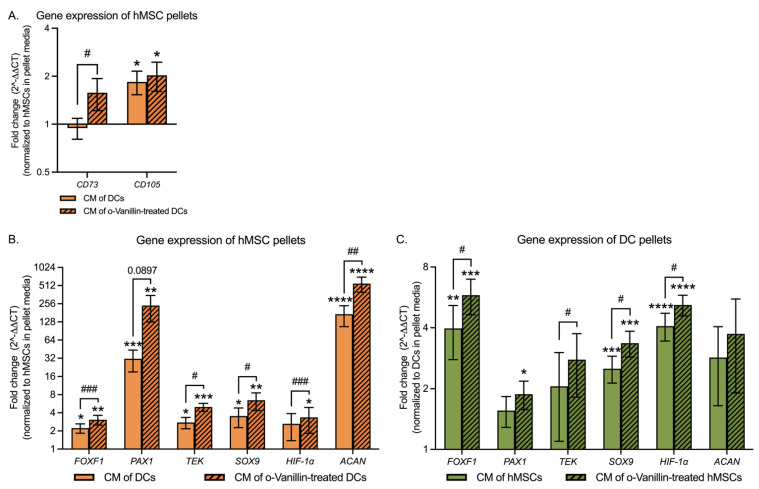
Gene expressions of hMSC and nucleus pulposus (NP) cell markers in hMSC and DC pellets with and without o-Vanillin treatment. (**A**) hMSC marker expressions in hMSC cultures exposed to CM of untreated and o-Vanillin-treated DCs. *n* = 8. (**B**) NP cell marker expressions in hMSC cultures exposed to CM of untreated and o-Vanillin-treated DCs. *n* = 8. (**C**) NP cell marker expressions in DC cultures exposed to CM of untreated and o-Vanillin-treated hMSCs. *n* = 8. Data were normalized to pellets cultured with regular pellet culture media and were not exposed to CM (control pellets). Values are presented as mean ± SEM. *, **, ***, and **** indicate a statistical significance of *p* < 0.05, *p* < 0.01, *p* < 0.001, and *p* < 0.0001, respectively, assessed by a paired *t-*test compared to the control pellets. #, ##, and ### indicate a statistically significant change of *p* < 0.05, *p* < 0.01, and *p* < 0.001 assessed by a paired *t-*test between untreated and o-Vanillin-treated pellets. The patterned histograms represent the group exposed to the CM of o-Vanillin-treated pellets. Additionally, see Table 2.

To evaluate the effect of vesicular transfer and o-Vanillin treatment on gene expression of known hMSC markers and specific NP mature and progenitor markers, qPCR was performed. Gene expression of *CD73* showed no change (0.05 ± 0.14-fold, *p* = 0.3969), whereas expression of *CD105* (0.84 ± 0.31-fold, *p* = 0.0169) significantly increased in hMSCs exposed to DC CM (Figure 5A). When exposed to o-Vanillin-treated DC CM, the expression of *CD73* also showed no change (0.58 ± 0.36-fold, *p* = 0.1648) compared to the control pellets, but was significantly increased by 0.64 ± 0.23-fold (*p* = 0.0146) compared to hMSCs exposed to DC CM. The expression of *CD105* (1.03 ± 0.42-fold, *p* = 0.0239) significantly increased compared to the control pellets, while it was not significantly changed (0.11 ± 0.13-fold, *p* = 0.6911) compared to hMSCs exposed to DC CM (Figure 5A).

NP mature and progenitor markers were also measured in hMSC and DC pellets to evaluate the effect of DC-derived EVs on hMSC differentiation toward an NP-like phenotype, and on the regenerative effect of hMSC-derived EVs on DCs. In addition, hMSC and DC pellets exposed to o-Vanillin-treated DC and hMSC pellet CM were evaluated to determine the effect of o-Vanillin treatment of the host cells. The expression of *FOXF1*, *PAX1*, *TEK (*also known as *TIE2*) (angiopoietin-1 receptor, also known as CD202B), *SOX9*, and *ACAN* increased significantly by 1.23 ± 0.39-fold (*p* = 0.0103), 30.26 ± 12.24-fold (*p* = 0.0005), 1.76 ± 0.59-fold (*p* = 0.0127), 2.54 ± 1.27-fold (*p* = 0.0327), and 170.8 ± 65.3-fold (*p* < 0.0001), respectively, in hMSCs exposed to DC CM, while the change of *HIF-1α* (1.63 ± 1.25-fold, *p* = 0.1326) expression was not significantly affected (Figure 5B). When exposed to o-Vanillin-treated DC CM, the expression of *FOXF1*, *PAX1*, *TEK*, *SOX9*, *HIF-1α*, and *ACAN* increased significantly by 2.07 ± 0.57-fold (*p* = 0.0015), 238.34 ± 111.39-fold (*p* = 0.0011), 3.99 ± 0.75-fold (*p* = 0.0001), 5.45 ± 2.11-fold (*p* = 0.0004), 2.36 ± 1.54-fold (*p* = 0.0275), and 551.15 ± 155.80-fold (*p* < 0.0001), respectively, in hMSCs compared to the control pellets (Figure 5B). Furthermore, the expression of *FOXF1* (0.38 ± 0.07-fold, *p* = 0.0005), *TEK* (1.46 ± 0.73-fold, *p* = 0.0229), *SOX9* (1.20 ± 0.70-fold, *p* = 0.0231), *HIF-1α* (0.34 ± 0.05-fold, *p* = 0.0001), and *ACAN* (4.51 ± 2.11-fold, *p* = 0.0076) was significantly increased by o-Vanillin treatment, while no statistically significant difference was found in *PAX1* (22.06 ± 18.99-fold, *p* = 0.0897) in hMSCs exposed to CM of o-Vanillin-treated DCs compared to those of hMSCs exposed to DC CM (Figure 5B).

In DCs exposed to hMSC CM, the expression of *FOXF1*, *SOX9*, and *HIF-1α* increased significantly by 2.97 ± 1.19-fold (*p* = 0.0097), 1.51 ± 0.38-fold (*p* = 0.0006), and 3.08 ± 0.64-fold (*p* < 0.0001), respectively, while the expression of *PAX1* (0.56 ± 0.27-fold, *p* = 0.1912), *TEK* (1.06 ± 0.96-fold, *p* = 0.3914), and *ACAN* (1.85 ± 1.20-fold, *p* = 0.2169) was not significantly changed (Figure 5C). When exposed to o-Vanillin-treated hMSC CM, the expression of *FOXF1*, *PAX1*, *SOX9*, and *HIF-1α* increased significantly by 4.80 ± 1.16-fold (*p* = 0.0002), 0.88 ± 0.30-fold (*p* = 0.0118), 2.35 ± 0.49-fold (*p* = 0.0004), and 4.19 ± 0.61-fold (*p* < 0.0001), respectively, while *TEK* (1.78 ± 0.96-fold, *p* = 0.0894) and *ACAN* (2.73 ± 1.83-fold, *p* = 0.3248) showed no changes in DCs compared to the control pellets (Figure 5C). Additionally, the expression of *FOXF1* (0.99 ± 0.44-fold, *p* = 0.0294), *TEK* (0.53 ± 0.22-fold, *p* = 0.0344), *SOX9* (0.36 ± 0.14-fold, *p* = 0.0287), and *HIF-1α* (0.35 ± 0.14-fold, *p* = 0.0326) was significantly increased by o-Vanillin treatment, while no statistically significant difference was found in *PAX1* (0.51 ± 0.46-fold, *p* = 0.2750) and *ACAN* (0.09 ± 0.18-fold, *p* = 0.9724) in DCs exposed to CM of o-Vanillin-treated hMSCs compared to those of DCs exposed to hMSC CM (Figure 5C).

These results indicate that DC CM with EVs contribute to hMSC differentiation toward NP-like cells, and that hMSC CM with EVs improve DC phenotypes. The effects were further improved by o-Vanillin treatment of the host cells.

## 4. Discussion

This study investigated the potential of o-Vanillin in improving hMSC-based therapy for IVD repair. The results confirmed previous studies showing that hMSCs enhance GAG production and deposition in co-cultures with DCs [35,36,37]. Pellets constructed at a 1:1 ratio of hMSCs and DCs resulted in the most robust GAG production and cell phenotypes, indicating that there is an optimal ratio of hMSCs to DCs for regenerative purposes. The cell density in human NP tissue is estimated between 2204 ± 636 and 2783 ± 2007 cells/mm^3^ (mean ± SD) in adults [38]. Given sufficient nutrition in the NP, 2200–2700 cells/mm^3^ might be a candidate density range of hMSCs for adult patients. Estimating the volume of NP and iAF compartments in a human IVD is 4 cm × 2.5 cm × 1 cm, the candidate hMSC dose may range from 22 × 10^6^ to 27 × 10^6^ cells. The doses of implanted hMSCs are highly variable in published studies. For example, Orozco et al. and Elabd et al. implanted 10 ± 5 × 10^6^ and 15.1–51.6 × 10^6^ (with autologous platelet lysate) autologous MSCs, respectively, in patients in 18–65 years of age with chronic LBP [39,40]. In addition, Noriega et al. implanted 25 × 10^6^ allogenic MSCs in patients in 18–75 years of age with chronic LBP [41]. Our finding may provide some guidance for the concentration of hMSCs for future work.

Co-culture of DCs with hMSCs reduced the number of senescent cells after 21 days in the pellets. This could be explained by an increased secretion of trophic factors from the hMSCs that restored and rejuvenated the disc cell phenotype. o-Vanillin is known to remove senescent IVD cells, and the data demonstrated that it also has senolytic effects on senescent hMSCs. o-Vanillin has senolytic effects as well as anti-inflammatory properties. Suppression of the inflammatory microenvironment in degenerating IVDs may further improve GAG deposition and disc cell phenotypes.

Cell transplantation carries some risks such as competition for the low nutrient and oxygen supply in the center of a human IVD. Degenerating IVDs possess progenitor cells; therefore, if the microenvironment could be improved, it would allow them to regenerate, thereby avoiding the need for cell transplantation. To investigate the potential for a cell-free system EV production and transfer between hMSCs and DCs was evaluated. Here, qualitative and quantitative data show that o-Vanillin promoted EV formation and/or uptake by both hMSCs and DCs.

The results demonstrated that hMSCs differentiated toward disc-like cells when exposed to DC CM in the absence of a discogenic differentiation media [42] or direct cell–cell contact. This contrasts the findings from Richardson et al., which showed that only co-culture with direct cell–cell contact of monolayer hMSCs resulted in differentiation toward a disc-like phenotype [43]. They did not detect changes of NP markers in DCs or hMSCs in co-cultures without cell–cell contact [43]. A difference is that here hMSC differentiation in 3D pellet cultures was examined. Studies showed that 3D culture improve cell–cell contact and cell-ECM interactions in hMSCs [44] and degenerate NP cells [45]. This may be because the 3D microenvironment is different than that of a monolayer culture, thereby influencing signalling activity to better support hMSC differentiation. The culture time is another important difference: DC CM was applied to hMSC pellets for 21 days. Moreover, the cell types in the work of Richardson et al. were different. They used NP tissues of an 18-year-old donor showing no evidence of degeneration [43], whereas here tissues from degenerating IVDs (Thompson scale IV-V) [46] of older individuals (42.6 ± 11.9 (mean ± SD)) undergoing spinal surgery for LBP were used. Cells of symptomatic degenerating IVDs are different from that of non-degenerating IVD tissues [47,48], which may affect hMSC differentiation in a different way. In addition, they focused on genes involved in ECM production, including *COL2A1*, *ACAN*, *VCAN*, *ELN*, and *SOX9*, in NP and hMSC co-cultures with and without direct cell–cell contact. Whereas *ACAN*, *SOX9*, *HIF-1α*, *FOXF1*, *PAX1*, and *TEK* in DC and hMSC pellets with CM transfer were evaluated in the current study.

Clinical studies have demonstrated that hMSC implantation attenuates discogenic LBP [39,40,41]; however, several limitations remain to be overcome. One factor is the hostile inflammatory environment found in degenerating IVDs, which may affect stem cell health and retention [49] and could polarize hMSCs into a proinflammatory phenotype [50]. Implanting a large number of hMSCs may also exhaust nutrient and oxygen for the native cells. Enriched and purified EVs or secretome generated *ex vivo* in a controlled environment, may overcome some of these shortcomings as the cell-free therapy provides therapeutic molecules with less immunogenicity [51]. Other advantages include longer shelf life and storage time, simpler and more feasible transportation, and less risk of tumorigenesis [52,53]. However, further investigation is necessary to standardize the criteria of cell-based and cell-free therapies.

Treating EV donor cells with o-Vanillin, a senolytic and anti-inflammatory natural compound, improved hMSC differentiation and reduced hMSC senescence. We have previously demonstrated that o-Vanillin can remove senescent IVD cells and improve tissue heath in intact human IVDs [17]. The findings presented here further support the senolytic activity of o-Vanillin while demonstrating its potential in stem cell therapy. Senescent cells are metabolically very active [54] and removing them would provide more nutrients to the native cells and perhaps better support hMSC retention and differentiation. In addition, removing the senescent-associated secreted factors which contribute to the inflammatory environment caused by senescent cells could prevent hMSC exhaustion. It remains to be determined if EV supplementation can provide equal or better results compared to hMSC transplantation *in vivo*.

## 5. Conclusions

The findings presented here suggest that o-Vanillin improves trophic effects of hMSCs and promotes hMSC differentiation toward a disc-like cell phenotype. This was evident in direct and indirect 3D pellet cultures of hMSCs and DCs from degenerate IVDs. The data indicate that the senolytic and anti-inflammatory properties of o-Vanillin have the potential to improve stem cell-based therapies. Enriched and purified EVs or secretome generated ex vivo in a controlled environment, may overcome some of the shortcomings of cell-based therapies. The advantages of lower immunogenicity, extended shelf life and storage time, simpler and more feasible transportation, and lower tumorigenesis risk make the cell-free therapy a promising treatment in IVD repair.

## Figures and Tables

**Table 1:** Patient information and applications.

				Experiments										
Donor	Age	Gender	Disc pathology	Levels	Safranin-O staining	DMMB assay ^1^	p16*^INK4a^* staining	ELISA ^2^	Fluorescent staining				qPCR ^5^	Hoechst assay
									Membrane signal transfer (Co-culture)	EV ^3^ identification (Confocal microscope)	Membrane signal transfer (CM ^4^)	EV quantification (Nano-Flow cytometry)		
1	32	M	Degenerative disc disease with an annular tear	L5-S1		✔	✔	✔						
2	59	F	Simple herniation	L5-S1		✔		✔						
3	38	F	Simple herniation	L5-S1	✔	✔		✔	✔				✔	
4	45	M	Degenerative disc disease	L5-S1	✔	✔	✔	✔	✔					
5	35	F	Degenerative disc disease	L4-5	✔	✔	✔	✔	✔					
6	42	F	Recurrent hernia	L5-S1				✔			✔			
7	51	F	Degenerative disc disease	L5-S1					✔	✔		✔	✔	✔
8	38	F	Degenerative disc disease	L4-S1										✔
9	49	F	Degenerative disc disease	L5-S1										✔
10	40	F	Degenerative disc disease	L5-S1										✔
11	26	M	Degenerative disc disease	L4-5	✔		✔				✔			✔
12	39	F	Back pain	L4-S1						✔	✔			
13	54	M	Back pain	L4-5						✔			✔	
14	44	F	Back pain	L4-5									✔	
15	23	F	Disc herniation	L5-S1									✔	
16	35	F	Degenerative disc disease	L4-S1										
17	54	M	Degenerative disc disease	L4-S1									✔	
18	50	F	Spinal stenosis/Spondylo	L5-S1									✔	
19	71	F	Simple herniation	L3-S1								✔		
20	45	F	Simple herniation	L4-S1								✔	✔	
21	24	F	Simple herniation	L4-5								✔		

^1^ DMMB assay: dimethyl methylene blue assay. ^2^ ELISA: enzyme-linked immunosorbent assay. ^3^ EV: extracellular vesicle. ^4^ CM: conditioned media. ^5^ qPCR: real-time quantitative polymerase chain reaction.

**Table 2:** qPCR primer list.

Target Gene	Name/Gene ID	Forward Primer Sequence (5′-3′)	Reverse Primer Sequence (5′-3′)	Primer Bank ID	Reference
*beta-ACTIN* (Reference gene)	*ACTB*/ID: 60	GTC TTC CCC TCC ATC GTG G	AAT CCT TCT GAC CCA TGC C		[29]
*CD73*	*NT5E*/ID: 4907	CCA GTA CCA GGG CAC TAT CTG	TGG CTC GAT CAG TCC TTC CA	325651882c2	
*CD105*	*ENG*/ID: 2022	TGC ACT TGG CCT ACA ATT CCA	AGC TGC CCA CTC AAG GAT CT	168693646c1	
*FOXF1*	*FOXF1*/ID: 2294	GCG GCT TCC GAA GGA AAT G	CAA GTG GCC GTT CAT CAT GC	110735444c1	
*PAX1*	*PAX1*/ID: 5075	TCG CTA TGG AGC AGA CGT ATG	GCT GCC GAC TGA TGT CAC A	380036025c1	
*TEK*	*TEK*/ID: 7010	TTA GCC AGC TTA GTT CTC TGT GG	AGC ATC AGA TAC AAG AGG TAG GG		[30]
*SOX9*	*SOX9*/ID: 6662	AGC GAA CGC ACA TCA AGA C	CTG TAG GCG ATC TGT TGG GG	182765453c1	
*HIF-1* *⍺*	*HIF1A*/ID: 3091	AAG GAA CCT GAT GCT TTA ACT TTG	TGG TCA TCA GTT TCT GTG TCG		[31]
*ACAN*	*ACAN*/ID: 176	TCG AGG ACA GCG AGG CC	TCG AGG GTG TAG CGT GTA GAG A		[32]

Cycling number: 40; Annealing temperature: 60 °C for all primers.

## Data Availability

All data generated or analyzed during this study are included in the manuscript and Supporting Files. The raw data and materials used to support the findings of this study are available from the corresponding author upon request.

## Supplementary Materials

The following supporting information can be downloaded at: https://www.mdpi.com/article/10.3390/cells11223589/s1, Figure S1: Schematic representation of the experimental design; Figure S2: qPCR sample setup; Figure S3: Metabolic activity of hMSC pellets and the cell loss and proliferation in untreated and o-Vanillin-treated pellets in the start and end of the 21-day culture. Related to Figure 1; Figure S4: EVs in complete cell culture media and from unlabeled hMSCs and DCs in the respective CM. Related to Figure 3; Figure S5: Unlabeled and labeled DC and hMSC control samples. Related to Figure 4. References [17,55] are cited in the Supplementary Materials.
