# Supplementary material for: o-Vanillin Modulates Cell Phenotype and Extracellular Vesicles of Human Mesenchymal Stem Cells and Intervertebral Disc Cells
Source: Cells. 2022 Nov 13;11(22):3589. doi: 10.3390/cells11223589 (PMC9688801; doi:10.3390/cells11223589)
Supplement: Supplementary file 1 [file cells-11-03589-s001.zip › cells-2001092-supplementary.pdf]

Supplementary figures

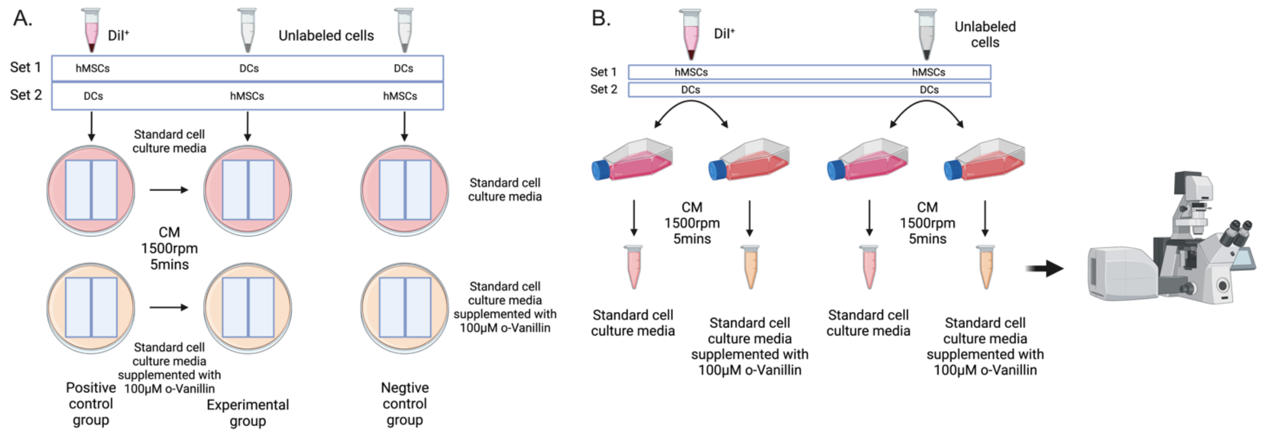

Figure S1. Schematic representation of the experimental design.

(A) EV production and uptake.

(B) The structure and size detection of EVs.

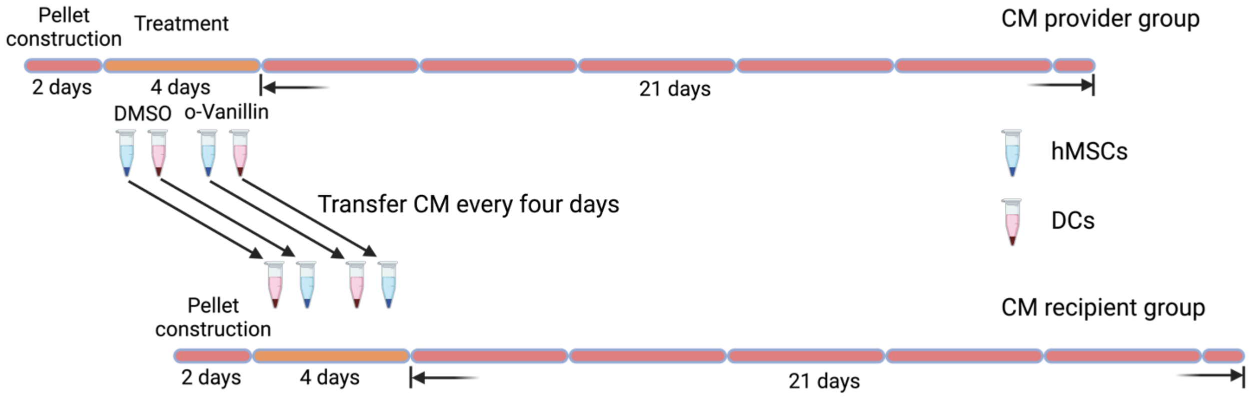

Figure S2. qPCR sample setup.

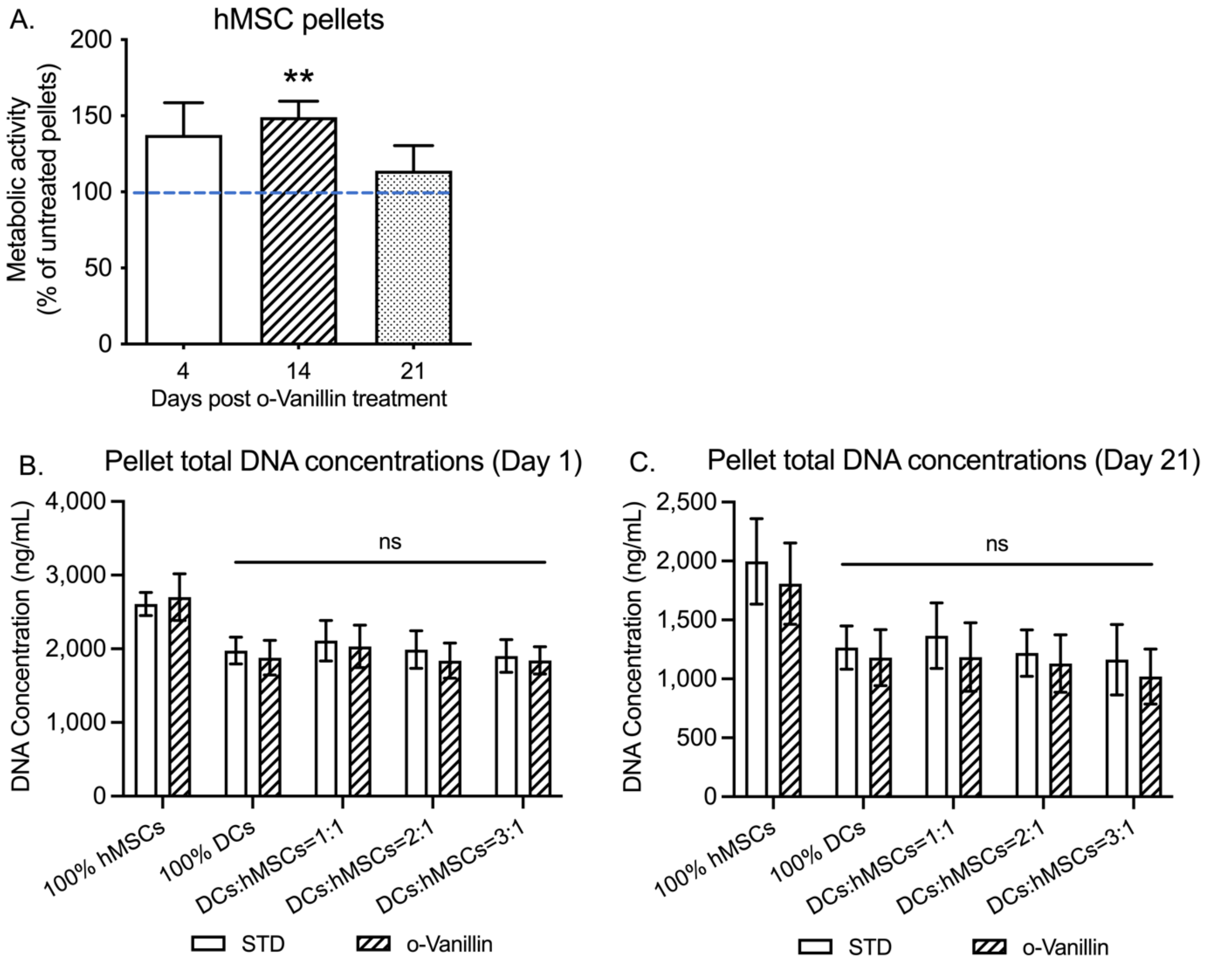

Figure S3. Metabolic activity of hMSC pellets and the cell loss and proliferation in untreated and o-Vanillin-treated pellets in the start and end of the 21-day culture. Related to **Figure 1**.

(A) Metabolic activities of hMSCs in day 4, 14, and 21 post treatment of o-Vanillin. Data is normalized to untreated hMSC pellets. $n = 3$.

(B and C) Quantification of total DNA concentrations in 100% hMSCs, 100% DCs, and co-cultures (1:1, 2:1, and 3:1 (DCs:hMSCs)) in Day 1 **(B)** and Day 21 **(C)**. $n = 5$.

Values are presented as mean ± SEM. ** indicates a statistically significant change assessed by a paired *t*-test: $p < 0.01$.

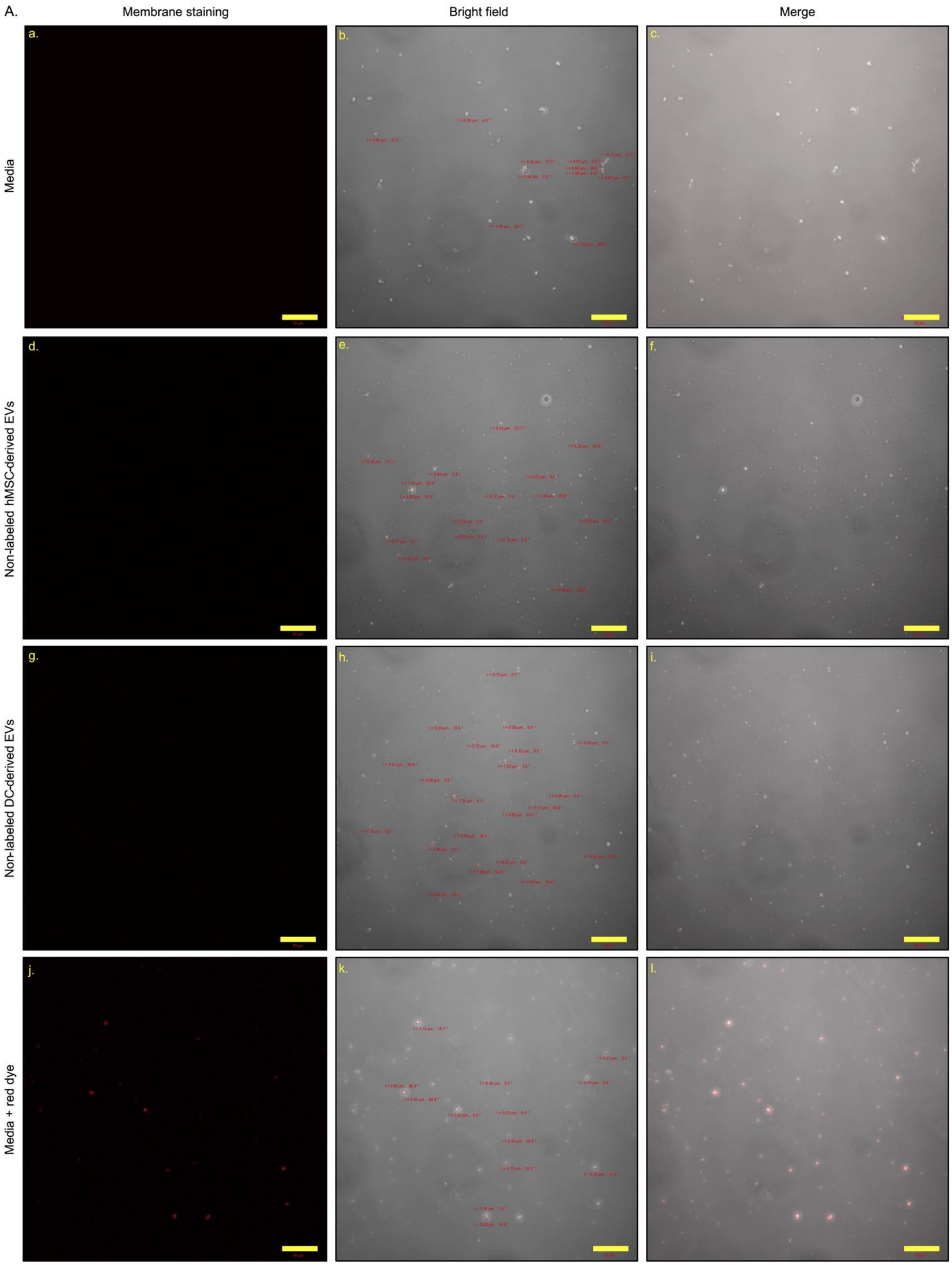
A.
Membrane staining
Bright field
Merge
Media
Non-labeled hMSC-derived EVs
Non-labeled DC-derived EVs
Media + red dye
a.
b.
c.
d.
e.
f.
g.
h.
i.
j.
k.
l.

Figure S4. EVs in complete cell culture media and from unlabeled hMSCs and DCs in the respective CM. Related to **Figure 3**.

A (a-c). Representative images showing EVs in cell culture media from FBS. No red signal was detected in the regular complete cell culture media (**A (a)**). A small quantity of bovine EVs from FBS was observed in the bright field (**A (b-c)**). **A (d-i).** Representative images showing the EV structures generated by unlabeled hMSCs (**d-f**) and DCs (**g-i**). No red signal was detected in the unlabeled hMSC CM (**A (d)**) and DC CM (**A (g)**). The EVs from hMSCs (**A (e-f)**) and DCs (**A (h-i)**) were observed in the bright field. **A (j-l).** Representative images showing EVs from FBS in cell culture media stained by DiI. The bovine serum EVs presented weak red signal when exposed to DiI in CM (**A (j and l)**). EV diameters were measured (**b, e, h,** and **k**). Scale bars: 10 µm. *n* = 3.

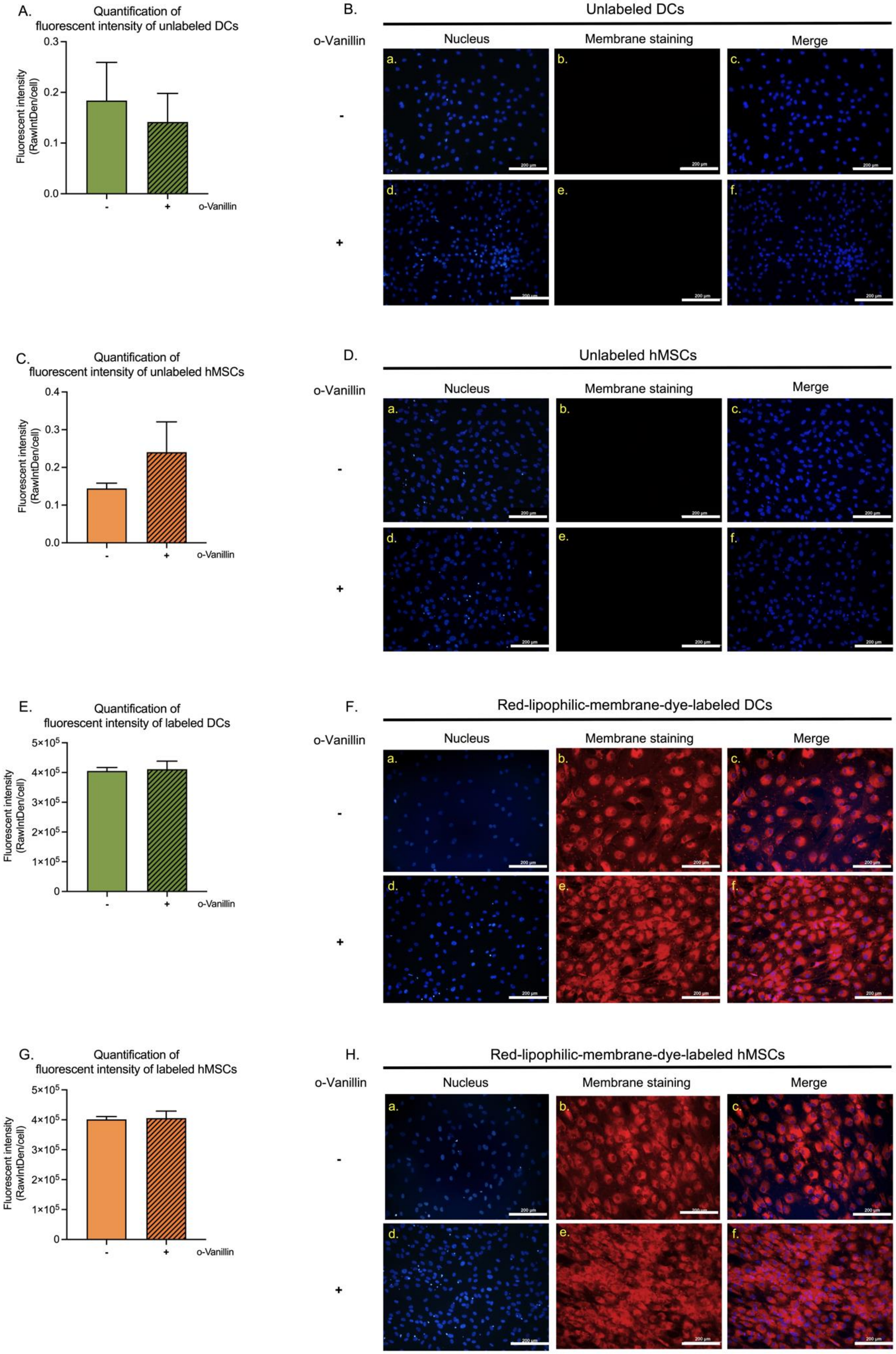
A.
Quantification of
fluorescent intensity of unlabeled DCs
Fluorescent intensity
(RawIntDen/cell)
0.3
0.2
0.1
0.0
-
+
o-Vanillin
B.
Unlabeled DCs
o-Vanillin
Nucleus
Membrane staining
Merge
a.
b.
c.
d.
e.
f.
-
+
C.
Quantification of
fluorescent intensity of unlabeled hMSCs
Fluorescent intensity
(RawIntDen/cell)
0.4
0.3
0.2
0.1
0.0
-
+
o-Vanillin
D.
Unlabeled hMSCs
o-Vanillin
Nucleus
Membrane staining
Merge
a.
b.
c.
d.
e.
f.
-
+
E.
Quantification of
fluorescent intensity of labeled DCs
Fluorescent intensity
(RawIntDen/cell)
5×10^5
4×10^5
3×10^5
2×10^5
1×10^5
0
-
+
o-Vanillin
F.
Red-lipophilic-membrane-dye-labeled DCs
o-Vanillin
Nucleus
Membrane staining
Merge
a.
b.
c.
d.
e.
f.
-
+
G.
Quantification of
fluorescent intensity of labeled hMSCs
Fluorescent intensity
(RawIntDen/cell)
5×10^5
4×10^5
3×10^5
2×10^5
1×10^5
0
-
+
o-Vanillin
H.
Red-lipophilic-membrane-dye-labeled hMSCs
o-Vanillin
Nucleus
Membrane staining
Merge
a.
b.
c.
d.
e.
f.
-
+

Figure S5. Unlabeled and labeled DC and hMSC control samples. Related to **Figure 4**.

(A) Quantification of cellular fluorescent intensities of unlabeled DCs cultured in standard pellet culture media with and without o-Vanillin treatment. $n = 3$.

(B) Representative images of unlabeled DCs cultured in standard pellet culture media without (**a-c**) and with (**d-f**) o-Vanillin treatment. Scale bars: 200 μm. $n = 3$.

(C) Quantification of cellular fluorescent intensities of unlabeled hMSCs cultured in standard pellet culture media with and without o-Vanillin treatment. $n = 3$.

(D) Representative images of unlabeled hMSCs cultured in standard pellet culture media without (**a-c**) and with (**d-f**) o-Vanillin treatment. Scale bars: 200 μm. $n = 3$.

(E) Quantification of cellular fluorescent intensities of labeled DCs cultured in standard pellet culture media with and without o-Vanillin treatment. $n = 3$.

(F) Representative images of labeled DCs cultured in standard pellet culture media without (**a-c**) and with (**d-f**) o-Vanillin treatment. Scale bars: 200 μm. $n = 3$.

(G) Quantification of cellular fluorescent intensities of labeled hMSCs cultured in standard pellet culture media with and without o-Vanillin treatment. $n = 3$.

(H) Photomicrographs of labeled hMSCs cultured in standard pellet culture media without (**a-c**) and with (**d-f**) o-Vanillin treatment. Scale bars: 200 μm. $n = 3$.

Representative images showing nuclear (blue), plasma membrane (red), and merge channels of unlabeled and labeled DCs and hMSCs. Patterned histograms represent o-Vanillin-treated groups. Values are presented as mean ± SEM. A paired *t*-test was conducted in (**A**), (**C**), (**E**), and (**G**). A statistically significant change was set as $p < 0.05$. There was no statistically significant change in (**A**), (**C**), (**E**), and (**G**).

Supplementary materials and methods

Metabolic activity

o-Vanillin's effect on hMSCs in pellet cultures was evaluated via metabolic activity using alamarBlue® Assay [1]. The assessments were performed at three time points: day 4, 14 and 21. As reported previously, no cytotoxic effect was observed for o-Vanillin at 100 μM on DCs [1]. For hMSCs, pellets were constructed for two days and then exposed to 100 μM o-Vanillin or vehicle 0.01% (v/v) DMSO for four days followed by an incubation in standard pellet culture media. In each assessment, 10% (v/v) alamarBlue® reagent (ThermoFisher Scientific, USA) was added to each sample and incubated for 6 hours at 37°C and 5% CO_2. Fluorescence was measured at excitation 560 nm and emission 590 nm using a TECAN Infinite M200 PRO plate reader with i-control 1.9 Magellan software (TECAN, Switzerland). Related to **Figure 1**.

Total DNA quantification

DNA quantification was assessed by a Hoechst Assay (Hoechst 33258, ThermoFisher Scientific, Toronto, ON, Canada) as described previously [2]. Samples were analyzed using a TECAN Infinite M200 PRO plate reader with i-control 1.9 Magellan software (TECAN, Männedorf, Switzerland). Related to **Figure 1**.

Supplementary references

1. Cherif, H.; Bisson, D.G.; Jarzem, P.; Weber, M.; Ouellet, J.A.; Haglund, L. Curcumin and o-Vanillin Exhibit Evidence of Senolytic Activity in Human IVD Cells In Vitro. *Journal of clinical medicine* **2019**, *8*, 433. doi:10.3390/jcm8040433.
2. Fairag, R.; Rosenzweig, D.H.; Ramirez-Garcialuna, J.L.; Weber, M.H.; Haglund, L. Three-Dimensional Printed Polylactic Acid Scaffolds Promote Bone-like Matrix Deposition in Vitro. *ACS applied materials & interfaces* **2019**, *11*, 15306-15315, doi:10.1021/acsami.9b02502.
